# Effects of combined use of compound acidifiers and plant essential oils in feed on the reproductive performance and physiological status of Xianjv chickens

**DOI:** 10.1016/j.psj.2024.104710

**Published:** 2024-12-20

**Authors:** Zhaobin Wang, Caiyun Zhang, Huicheng Wang, Tiantian Gu, Yong Tian, Zhuoya Gu, Li Chen, Tao Zeng, Lizhi Lu, Wenwu Xu

**Affiliations:** a State Key Laboratory for Managing Biotic and Chemical Threats to the Quality and Safety of Agro-products, Key Laboratory of Livestock and Poultry Resources (Poultry) Evaluation and Utilization, Ministry of Agriculture and Rural Affairs, Institute of Animal Science & Veterinary, Zhejiang Academy of Agricultural Sciences, Hangzhou 310021, PR China; b Xianghu Laboratory, Hangzhou, 311231, PR China; c College of Animal Science and Technology, Anhui Agricultural University, Hefei 230061, PR China; d College of Animal Science and Technology, Northeast Agricultural University, Harbin 150030, PR China; e College of Animal Science and Technology, Yangzhou University, Yangzhou 225009, PR China

**Keywords:** Compound acidifiers, Essential oils, Xianjv chicken, Production performance, Nutritional regulation

## Abstract

This study investigates the effects of combined compound acidifiers and plant essential oils on the production performance, egg quality, and health parameters of Xianjv chickens. A total of 240 healthy 34-week-old Xianjv chickens were randomly divided into 5 groups and given 5 different feed additives: a control group with a basal diet, and four experimental groups with varying doses of compound acidifiers (CA) and essential oils (EO). The results revealed that the addition of compound acidifiers and essential oils did not significantly affect average daily feed intake, egg production rate, or feed-to-egg ratio. However, supplementation with CA and EO significantly improved eggshell strength, albumen height, and Haugh unit, enhancing overall egg quality. Additionally, the combination of these additives enhanced serum antioxidant capacity by increasing levels of superoxide dismutase (SOD), glutathione peroxidase (GSH-Px), and catalase (CAT), while reducing malondialdehyde (MDA) levels. It also lowered inflammatory cytokines such as interleukin-2 (IL-2) and interleukin-6 (IL-6) and improved intestinal health by enhancing digestive enzyme activities, including trypsin and lipase, in the duodenum and jejunum. The study concludes that the combined use of compound acidifiers and plant essential oils offers a promising alternative to antibiotics in poultry feed, improving egg quality and supporting better health status in Xianjv chickens. Nevertheless, careful optimization of the dosages and combinations is necessary to maximize their benefits without adversely affecting the birds' physiological balance.

## Introduction

With the intensive development of agricultural practices, the problem of antibiotic residues has become increasingly serious, and antibiotic resistance, an emerging public health crisis, has become a point that cannot be ignored. Antibiotics are mainly used in animal husbandry as growth promoters to increase the growth rate of livestock and poultry, improve feed efficiency and reduce the incidence of livestock and poultry diseases. However, long-term use or abuse, along with inadequate withdrawal period control, can lead to environmental pollution caused by antibiotic residues, which can harm livestock and poultry (Fei, et al., 2023; Hu and Cowling, 2020; Kyuchukova, 2020). Globally, the poultry industry is under pressure to replace antibiotic growth promoters with green and safe feed additives, which has prompted researchers to conduct large-scale exploration of the use of natural additives.

Essential oils (EO) are volatile substances extracted from plant materials mainly by steam distillation. They are mainly composed of dozens of complex mixture components, including a group of terpenoids (linalool, menthol, borneol, geraniol, α-terpineol) and a group of low molecular weight aliphatic hydrocarbons (thymol, eugenol, carvacrol, cinnamaldehyde), and are one of the most promising poultry growth promoters (Irawan, et al., 2021). The research on essential oils is widely used in broilers. The beneficial effects of essential oils are related to their regulation of multiple metabolic pathways, including stimulation of digestive enzyme secretion and activity, regulation of lipid metabolism, antimicrobial effects, and improvement of intestinal integrity in chickens (Brenes and Roura, 2010; Stevanović, et al., 2018). Additionally, studies have shown that essential oil supplements can effectively replace the use of antibiotic growth promoters (Aristimunha, et al., 2016). In laying hens, Ding et al. found that essential oil supplementation could significantly improve protein digestibility and increase eggshell thickness in laying hens (Ding, et al., 2017). On the contrary, some studies have shown that different levels of essential oil mixtures in the diet have no significant effect on laying hen production performance parameters, egg breakage, and eggshell weight (Olgun and Yıldız, 2014), which also led to inconsistent results.

Acidifiers are considered as another possible feed additive to replace antibiotics. Acidifiers can improve the digestibility of nutrients and play an important role in growth performance and animal intestinal health (Gao, et al., 2021). Acidifiers can lower the pH value of the gastrointestinal tract, inhibit the growth of harmful bacteria, and increase the activity of proteolytic enzymes. The inclusion of acidifiers in the feed helps maintain the optimal pH in the stomach for enzymatic action and ensures proper protein digestion in the intestine (Lückstädt and Mellor, 2011).

The Xianjv chicken originates from Xianjv County in Zhejiang Province. It is regarded as one of the most famous local egg-laying chicken breeds in China due to its high egg production (180 to 200 eggs per year) and strong adaptability (Tang, et al., 2009). Plant essential oils and acidifiers have the above-mentioned advantages, but there are still doubts about their compounding in Xianjv chicken feed. Therefore, this study mainly explores the effects of the combined use of compound acidifiers and plant essential oils on the production performance and intestinal health of Xianjv chickens, and provides a certain reference basis for the application of compound acidifiers in the production process of Xianjv chickens and whether the combination effect of compound acidifiers and plant essential oils is better than using a single compound acidifier.

## Materials and methods

### Animal ethics statement

Animal management and experimental procedures were completely carried out following the Guidelines for Care and Use of Laboratory Animals of Zhejiang Academy of Agricultural Sciences (Approval number: 2022ZAASLA59).

### Experimental materials and animals

The compound acidifier (CA) is encapsulated acid provided by Hangzhou Kangdequan Feed Co., Ltd., and its effective ingredients are fumaric acid ≥25.0 %, sorbic acid ≥8.0 %, citric acid ≥8.0 %, and malic acid ≥ 8.0 %. The plant essential oil (EO) is "Aikedaning" provided by Hangzhou Kangdequan Feed Co., Ltd., and its effective ingredients are cinnamaldehyde ≥ 5.0 %, carvacrol ≥ 8.0 %, and thymol ≥ 2.0 %. The Xianjv chicken used in this study is an excellent local breed of small-scale laying hens in my country. The experimental animals were 600 healthy Xianjv chickens of 34 weeks of age, similar in body condition, and similar in egg production. The experimental animals and experimental sites were provided by Xianjv Breeding Farm in Xianjv County, Taizhou City, Zhejiang Province.

### Experimental design and feeding management

The basic feed formula was prepared according to the Chinese Feed Composition and Nutritional Value Table (31^st^ Edition) and the national feeding standard for chickens (NY/T33-2004), combined with actual production needs. The composition and nutritional components are detailed in Table 1. A total of 240 Xianjv laying hens, aged 34 weeks, were randomly divided into 5 groups, each with 24 replicates of 2 hens per replicate. Group A served as the blank control group and was fed a basal diet. Group B, the low-dose compound group, received a basal diet supplemented with 100 mg/kg essential oils (EO) and 1 g/kg compound acidifiers (CA). Group C, the low-CA experimental group, received a basal diet supplemented with 1 g/kg CA. Group D, the high-dose compound group, received a basal diet supplemented with 100 mg/kg EO and 1.5 g/kg CA. Group E, the high-CA experimental group, received a basal diet supplemented with 1.5 g/kg CA. Before the start of the experiment, the chicken coop was thoroughly washed and disinfected. The experiment utilized a two-tiered stepped cage system, with two chickens per cage and four consecutive cages forming one replicate. The experimental chickens had free access to feed, and water was supplied through a nipple drinker system. An intelligent device controlled the lighting, temperature, humidity, and ventilation in the chicken coop, ensuring sufficient lighting from 5:00 AM to 8:00 PM for a total of 16 hours per day. Manual feeding ensured that each group received the same amount of feed as much as possible. Feed was added based on the chickens' consumption at 7:30 AM and 5:00 PM daily, and feed consumption was recorded. Egg production was recorded for each replicate at 9:00 AM daily, including the number of eggs laid and their weight, as well as the number of defective eggs such as broken or soft-shelled eggs. The health of the flock was regularly monitored, feed troughs were cleaned, and the coop was maintained, including regular disinfection and epidemic prevention to ensure good hygiene conditions. The pre-feeding period lasted 7 days, followed by a formal experimental period of 56 days.

**Table 1:** Composition and nutrient levels of basal diets (air-dry basis, %)

Feed composition	Content (%)	Nutritional Levels	Content (%)
Corn	55.00	AME (MJ/kg)	11.30
Soybean meal (CP 46 %)	25.00	CP	17.84
Rice bran	4.00	Ca	3.88
Fish Meal (CP 60 %)	3.00	Available P	0.42
Shell powder	3.00	Lys	0.87
Calcium phosphate	6.50	Met	0.32
Calcium dihydrogen phosphate	1.20	Thr	0.58
Salt	0.30	Trp	0.18
Premixes^1^	2.00		
**Total**	**100**		

Note:

1 Premix is provided per kg of diet: Vitamin A 1,500 IU, Vitamin B_1_ 1.5 mg, Vitamin B_2_ 3.5 mg, Vitamin B_3_ 10 mg, Vitamin B_6_ 3.0 mg, Vitamin B_12_ 10 μg, Vitamin D_3_ 200 IU, Vitamin E 10 IU, Vitamin H 0.15 mg, Niacin 30 mg. Choline 1,000 mg, Iron 80 mg, Zinc 40 mg, Manganese 60 mg, Iodine 0.18 mg, Copper 8 mg, Selenium 0.3 mg.

### Sample collection and Detection

#### Feed chemical composition analysis

Representative feed samples were collected for nutrient level analysis. Metabolizable energy (AME) was calculated by using Tables of Feed Composition and Nutritive Values in China (2020). Crude protein (CP) was analysed according to official method 990.03 (AOAC, 1995). Calcium was analysed according to official method 927.02 (AOAC, 1995). Available phosphorus was analysed according to official method 965.17 (AOAC, 1995). Lysine was analysed according to official method 988.15 (AOAC, 1990). Methionine, tryptophan and threonine was analysed according to official method 994.12 (AOAC, 1995).

#### Production performance measurement

During the trial, egg production and feed consumption of each replicate were recorded daily, and eggs were weighed. The number of broken eggs, soft-shell eggs, and other defective eggs were recorded for each replicate. Feed consumption, average daily feed intake (ADFI), total egg production, total egg weight, feed-to-egg ratio, and other production data were calculated during the trial. The calculation formulas for these indicators were as follows:

ADFI (g/day) = (Total feed intake (g) / experiment days (d)) / Number of hens per replicate;
The average egg weight (g) = Total egg weight per replicate (g)/Number of laying hens per replicate;
Egg production rate (%) = (Total number of eggs produced per replicate / number of laying hens per replicate) × 100%;
feed-to-egg ratio = Average daily feed intake (g) /Average egg weight (g).

#### Egg quality determination

At the end of the experiment, 10 eggs with similar shapes and no shell contamination or damage were selected from each replicate. Egg quality measurements were conducted within 12 hours. The egg shape index was measured using a vernier caliper (Shanghai Jiuliang Hardware Tools Co., Ltd., with a measurement accuracy of 0.01 mm). An ORKA egg quality automatic analyzer (Model EA-01, ORKA Company, Israel) was used to measure egg weight, albumen height, Haugh unit, and yolk color. Eggshell strength was measured using an eggshell strength tester (Model III, Robotmation Co., Japan). Eggshell thickness was measured using an eggshell thickness analyzer (Model 1061, Robotmation Co., Japan). The calculation formula is as follows:

Egg shape index = egg length (mm) / egg width (mm).

#### Measurement of serum biochemical parameters, cytokines, antioxidant parameters and hormone levels

After the experiment, one laying hen with a body weight close to the average was randomly selected from each replicate. After a 12-hour fast, 5 mL of blood was collected from the jugular vein. The blood was left at room temperature for 1 hour until serum separates. The serum was then centrifuged at 3000 rpm, and the serum was carefully drawn off with a pipette and transferred to a 1.5 mL sterile Eppendorf tube. The samples were stored in a -20°C freezer until analysis. Total protein (TP), albumin (ALB), high-density lipoprotein (HDL), low-density lipoprotein (LDL), immunoglobulin A (IgA), immunoglobulin G (IgG), immunoglobulin M (IgM), and other serum biochemical parameters were detected using the Mindray BS-420 automatic biochemistry analyzer. The levels of superoxide dismutase (SOD), glutathione peroxidase (GSH-Px), total antioxidant capacity (T-AOC), catalase (CAT), and malondialdehyde (MDA) were determined using assay kits. Progesterone (P), estrogen (E2), prolactin (PRL), follicle-stimulating hormone (FSH), luteinizing hormone (LH), growth hormone (GH), triiodothyronine (T3), thyroxine (T4), IL-2, and IL-6 were measured using the ELISA method. All assay kits were purchased from Beijing Huaying Biotechnology Research Institute, and the reagent preparation and operations were carried out according to the instructions provided.

#### Measurement of intestinal digestive enzyme activity

The activities of trypsin, amylase, and lipase in the duodenum and jejunum were determined using corresponding assay kits according to the manufacturer's instructions. The assay kits were purchased from Nanjing Jiancheng Bioengineering Institute (Nanjing, China).

### Statistical analysis

The data were subjected to one-way analysis of variance using SPSS 20.0 (SPSS Inc., USA), and Turkey's post hoc test was performed. All results were presented as mean ± SEM. The same or no superscripts within a row indicated no significant difference (*P*>0.05), different superscripts within a row indicated a significant difference (*P*<0.05).

## Results

### Production performance

As shown in Table 2, the addition of a compound acidifier and its combination with plant essential oils in the feed had no significant effect on the average feed intake, egg production rate, average egg weight, and feed-to-egg ratio of Xianjv chickens during the entire experimental period (*P*>0.05).

**Table 2:** Effect of composite acidifier and its compound with plant essential oil on the production performance of Xianjv chickens

Items	Group A	Group B	Group C	Group D	Group E
**Week 1 to 8**					
Average daily feed intake, g/d	96.84±1.22	96.83±0.35	96.67±2.12	96.97±0.52	96.01±0.34
Egg laying rate, %	63.82±5.94	66.76±4.37	65.47±3.56	67.60±4.41	66.93±1.15
Average egg weight, g	48.19±1.00	47.39±1.55	47.47±0.60	47.21±1.46	47.65±1.16
Feed to egg ratio	2.01±0.04	2.05±0.08	2.04±0.34	2.06±0.07	2.01±0.07

Note: The results were expressed as “mean ± SEM”. Group A (the control group), fed with basal feed; Group B, fed with 100 mg/kg essential oil (EO) and 1 g/kg compound acidifiers (CA); Group C, fed with 1 g/kg CA; Group D, fed with 100 mg/kg EO and 1.5 g/kg CA; Group E, fed with 1.5 g/kg CA. The same or no superscripts within a row indicate no significant difference (*P*>0.05), different superscripts within a row indicate a significant difference (*P*<0.05).

### Egg quality

As shown in Table 3, the eggshell strengths of Groups C and D were significantly higher than that of the control group (Group A) (*P*<0.05). The albumen height and Haugh unit of Groups C, D, and E were significantly higher than those of the control group (Group A) (*P*<0.05). There was no difference in eggshell thickness, yolk color, and egg shape index among the groups (*P*>0.05).

**Table 3:** Effect of compound acidifier and its combination with plant essential oil on the egg quality of Xianjv chickens

Items	Group A	Group B	Group C	Group D	Group E
Shell strength, g/cm^2^	4.08±0.54^c^	4.42±0.12^bc^	4.58±0.26^b^	5.00±0.17^a^	4.42±0.35^bc^
Shell thickness, mm	0.41±0.25	0.41±0.30	0.42±0.20	0.43±0.34	0.44±0.33
Albumen height, mm	4.33±0.44^b^	4.68±0.24^b^	5.23±0.32^a^	5.07±0.27^a^	5.18±0.18^a^
Yolk color	4.17±0.46	4.34±1.03	4.22±0.58	4.34±0.30	3.89±0.50
Haugh unit	67.92±3.13^c^	69.37±2.21^b^	72.09±1.89^ab^	72.53±1.76^a^	74.00±2.23^a^
Egg-shape index	1.29±0.04	1.28±0.03	1.29±0.08	1.30±0.02	1.31±0.02

Note: The results were expressed as “mean ± SEM”. Group A (the control group), fed with basal feed; Group B, fed with 100 mg/kg essential oil (EO) and 1 g/kg compound acidifiers (CA); Group C, fed with 1 g/kg CA; Group D, fed with 100 mg/kg EO and 1.5 g/kg CA; Group E, fed with 1.5 g/kg CA. The same or no superscripts within a row indicate no significant difference (*P*>0.05), different superscripts within a row indicate a significant difference (*P*<0.05).

### Serum biochemical parameters and cytokines

As shown in Table 4, compared with the control group (Group A), the TP content in Group D was significantly reduced (*P*<0.05), and the IgG contents in Groups B and D were significantly increased (*P*<0.05). The IgM contents in Groups B, C, and D were significantly increased (*P*<0.05). The levels of cytokines IL-2 and IL6 in Groups B, C, and D were significantly reduced (*P*<0.05). However, the IL-6 level in Group E was significantly increased (*P*<0.05).

**Table 4:** Effect of compound acidifier and its compounding with plant essential oil on the serum biochemistry of Xianjv chickens

Items	Group A	Group B	Group C	Group D	Group E
TP, g/L	41.71±15.02^a^	34.61±16.47^ab^	26.84±6.05^ab^	25.93±5.16^b^	33.95±4.06^ab^
ALB, g/L	10.08±4.80	12.72±4.60	11.99±3.97	14.45±2.98	12.56±3.25
HDL, mmol/L	0.67±0.43	0.75±0.46	0.62±0.34	0.81±0.25	0.74±0.28
LDL, mmol/L	0.62±0.50	0.67±0.43	0.58±0.46	0.98±0.75	0.90±0.57
IgA, g/L	1.37±0.25	1.63±0.35	1.50±0.19	1.70±0.16	1.44±0.32
IgG, g/L	2.15±0.10^b^	2.55±0.31^a^	2.46±0.39^ab^	2.67±0.28^a^	2.37±0.28^ab^
IgM, g/L	0.87±0.03^c^	1.23±0.20^ab^	1.12±0.17^b^	1.38±0.22^a^	1.13±0.10^bc^
IL-2, pg/mL	365.81±26.96^a^	318.82±14.48^b^	283.42±53.14^b^	220.52±23.80^c^	379.41±18.50^a^
IL-6, pg/mL	187.71±9.44^b^	169.75±5.56^c^	159.40±14.91^c^	95.97±6.91^d^	205.21±24.50^a^

**Note:** The results were expressed as “mean ± SEM”. Group A (the control group), fed with basal feed; Group B, fed with 100 mg/kg essential oil (EO) and 1 g/kg compound acidifiers (CA); Group C, fed with 1 g/kg CA; Group D, fed with 100 mg/kg EO and 1.5 g/kg CA; Group E, fed with 1.5 g/kg CA. TP: Total Protein; ALB: Albumin; HDL: High-density lipoprotein; LDL: Low-density lipoprotein; IgA: Serum Immunoglobulin A; IgG: Serum immunoglobulin G; IgM: Serum immunoglobulin M; IL-2: Interleukin 2; IL-6: Interleukin 6. The same or no superscripts within a row indicate no significant difference (*P*>0.05), different superscripts within a row indicate a significant difference (*P*<0.05).

### Serum antioxidant parameters

Based on Table 5, the differences in serum antioxidant parameters between the groups were significant (*P*<0.05). Compared to the control group (Group A), the levels of SOD, T-AOC, GSH-P, and CAT in Groups C and D were significantly increased (*P*<0.05), and the level of GSH-P in Group B was also significantly higher than in control group (Group A) (*P*<0.05). The MDA levels in Groups B, C, and D were significantly lower than those in control group (Group A) (*P*<0.05). The levels of GSH-P and CAT in Group E significantly decreased, while the MDA level significantly increased (*P*<0.05).

**Table 5:** Effect of compound acidifier and its combination with plant essential oil on the antioxidant capacity of Xianjv chickens

Items	Group A	Group B	Group C	Group D	Group E
SOD, U/mL	55.75±8.30^cd^	61.84±5.10^bc^	69.14±13.93^ab^	74.72±2.46^a^	50.05±7.56^d^
GSH-P, U/mL	227.20±14.72^d^	261.64±22.84^c^	301.89±25.14^b^	353.39±24.44^a^	157.59±63.44^e^
T-AOC, U/mL	8.52±0.28^cd^	8.99±0.58^c^	9.75±0.28^b^	10.73±0.60^a^	8.11±0.15^d^
CAT, U/mL	23.41±3.87^c^	27.13±2.97^c^	35.51±3.07^b^	45.06±3.33^a^	19.07±2.80^d^
MDA, U/mL	4.73±0.39^b^	4.21±0.42^c^	3.80±0.28^c^	2.57±0.43^d^	5.47±0.26^a^

**Notes:** The results were expressed as “mean ± SEM”. Group A (the control group), fed with basal feed; Group B, fed with 100 mg/kg essential oil (EO) and 1 g/kg compound acidifiers (CA); Group C, fed with 1 g/kg CA; Group D, fed with 100 mg/kg EO and 1.5 g/kg CA; Group E, fed with 1.5 g/kg CA. SOD: Superoxide dismutase; GSH-PX: Glutathione peroxidase; MDA: Malondialdehyd; CAT: Catalase; T-AOC: Total antioxidant capacity. The same or no superscripts within a row indicate no significant difference (*P*>0.05), different superscripts within a row indicate a significant difference (*P*<0.05).

### Reproductive hormone levels

Based on Table 6, there were no significant differences in serum hormones Progesterone, Triiodothyronine, and Thyroid hormone among the groups (*P* > 0.05). Compared to control group (Group A), the estradiol levels in groups B, C, and D were significantly increased (*P* < 0.05), while the follicle-stimulating hormone levels in group D were significantly decreased (*P* < 0.05). The luteinizing hormone levels in groups C, D, and E were lower than those in control group (Group A) (*P* < 0.05), and the growth hormone levels in group D were significantly higher than those in group A (*P* < 0.05). Additionally, we observed variations in hormone levels among the experimental groups (excluding the control group (Group A)). Due to the supplementation of 1.5 g/kg CA (group E), the estradiol level in the serum of laying hens in this group was significantly lower than that in group B and D (*P* < 0.05). The growth hormone level in group E also significantly decreased (*P* < 0.05).

**Table 6:** Effect of compound acidifier and its compounding with plant essential oil on reproductive hormones in Xianjv chickens

Items	Group A	Group B	Group C	Group D	Group E
P, ng/mL	7.53±2.37	9.64±4.56	8.73±4.16	6.67±3.32	8.08±2.91
E2, pg/mL	42.31±10.94^c^	58.17±12.56^b^	74.51±11.39^a^	64.17±5.19^ab^	30.53±10.11^c^
FSH, mIU/mL	7.66±0.52^b^	7.22±0.71^bc^	6.70±1.26^bc^	6.49±0.38^c^	8.41±0.35^a^
LH, mIU/mL	14.56±1.10^a^	12.87±2.42^a^	10.55±1.56^b^	9.09±1.09^b^	5.44±0.29^c^
GH, ng/mL	6.97±1.62^bc^	8.46±0.53^abc^	9.40±0.35^ab^	10.87±2.68^a^	6.01±3.79^c^
T3, ng/mL	1.28±0.12	1.24±0.04	1.23±0.07	1.20±0.07	1.32±0.12
T4, ng/mL	16.40±0.60	15.23±1.67	15.18±0.30	14.39±3.23	17.62±1.13

Note: The results were expressed as “mean ± SEM”. Group A (the control group), fed with basal feed; Group B, fed with 100 mg/kg essential oil (EO) and 1 g/kg compound acidifiers (CA); Group C, fed with 1 g/kg CA; Group D, fed with 100 mg/kg EO and 1.5 g/kg CA; Group E, fed with 1.5 g/kg CA. P: Progesterone; E2: Estradiol2; FSH: Follicle stimulating hormone; LH: Luteinizing hormone: GH Growth hormone; T3: Triiodothyronine; T4: Thyroid hormone. The same or no superscripts within a row indicate no significant difference (*P*>0.05), different superscripts within a row indicate a significant difference (*P*<0.05).

### Intestinal digestive enzyme activity

According to Table 7, there were no significant changes in amylase levels in the duodenum and jejunum of Xianjv chickens across all groups (*P*>0.05). In the duodenum, the levels of lipase and trypsin were significantly higher in Groups C, D, and E compared to the control group (Group A) (*P*<0.05). In the jejunum, lipase levels in Groups D and E were significantly higher than those in the control group (Group A) (*P*<0.05). Additionally, trypsin levels in the jejunum were significantly higher in Groups C and D compared to the control group (Group A) (*P*<0.05).

**Table 7:** Effect of compound acidifier and its combination with plant essential oil on intestinal digestive enzymes in Xianjv chickens

Items	Group A	Group B	Group C	Group D	Group E
**Duodenum**					
Amylase, U/mg.Prot	24.98±14.66	44.20±29.76	24.87±16.75	63.46±35.51	20.36±10.90
Lipase, U/mg.Prot	27.11±4.62^c^	30.66±3.69^bc^	34.76±2.63^ab^	37.82±6.56^a^	40.12±2.53^a^
Trypsin, U/mg.Prot	89.46±3.95^c^	98.66±11.93^bc^	115.02±16.62^ab^	123.16±18.21^a^	126.36±12.54^a^
**Jejunum**					
Amylase, U/mg.Prot	61.32±26.27	59.58±17.18	73.64±14.28	66.98±34.21	47.45±41.25
Lipase, U/mg.Prot	31.04±6.15^c^	36.58±3.45^c^	40.14±5.81^bc^	47.66±6.54^ab^	55.46±12.21^a^
Trypsin, U/mg.Prot	106.45±18.25^c^	125.94±19.19^bc^	138.18±4.82^ab^	155.93±15.69^a^	135.10±25.00^adc^

**Note:** The results were expressed as “mean ± SEM”. Group A (the control group), fed with basal feed; Group B, fed with 100 mg/kg essential oil (EO) and 1 g/kg compound acidifiers (CA); Group C, fed with 1 g/kg CA; Group D, fed with 100 mg/kg EO and 1.5 g/kg CA; Group E, fed with 1.5 g/kg CA. The same or no superscripts within a row indicate no significant difference (*P*>0.05), different superscripts within a row indicate a significant difference (*P*<0.05).

## Discussion

Numerous studies have focused on acidifiers, and essential oils as alternatives to antibiotics for improving poultry performance and gut health. However, few studies have investigated the combined use of these additives and their compound effects on poultry. Several recent studies have shown that plant essential oils improve feed utilization in broilers (Hu, et al., 2023) and laying hens (Xu, et al., 2024). The primary reason for this is that the terpenoids in essential oils can restore microbial balance in the avian digestive system and improve nutrient absorption, thereby producing beneficial effects (Abd El-Hack, et al., 2022). Acidifiers have similar effects in improving the growth performance of broiler chickens (Sedghi, et al., 2024). However, Acidifiers have different effects on the production performance of laying hens. Supplementing acidifiers had no effect on egg production rate, feed intake, feed conversion ratio, and eggshell breakage rate in laying hens (Gong, et al., 2021). In another study, a mixture of 150 mg/kg encapsulated essential oils and organic acids significantly improved the egg production rate of laying hens (Wang, et al., 2019). In this experiment, there were no significant changes in the average daily feed intake, feed conversion ratio, average egg weight, and egg production rate among the groups. The reason may be due to differences in breed or environmental factors. The type and ratio of compound acidifiers and essential oils added will also have different degrees of influence on the production performance of laying hens. Additionally, supplementing with plant essential oils and compound acidifiers, especially in the group with 100 mg/kg EO and 1.5 g/kg CA, significantly increased eggshell strength, albumen height, and Haugh unit in Xianjv chickens. This may be related to the increase in the number of probiotics in laying hens (Mas'ad, et al., 2020). A highly acidic stomach environment helps secrete more gastric juice and pancreatic enzymes (Herszényi, et al., 2019). From our existing results, we found that the combination of EO and CA significantly increased the digestive enzyme activities (especially Lipase and Trypsin) in the duodenum and jejunum of Xianjv chickens. Although there is currently insufficient evidence to show that acidifiers and plant essential oils are directly related to increased digestive enzyme levels, supplementation with acidifiers can reduce gastric pH to a certain extent, thereby inhibiting the proliferation of pathogenic bacteria, stimulating the growth and activity of beneficial intestinal bacteria and promoting the activity of digestive enzymes. These key factors contribute to the effective digestion of food and absorption of nutrients (Trifanov, et al., 2020), thereby improving egg quality.

As the feed and rearing environment change, blood biochemical parameters continuously reflect the animal's current health status and nutritional levels (Hou, et al., 2024). It was previously reported that 300 g of protected organic acids–essential oils blend/1,000 kg of feed treatment had no significant effect on total protein, albumin, and globulin in broiler serum (Adewole, et al., 2021). The total protein level is an important indicator reflecting the liver's protein synthesis metabolism (Yuan, et al., 2016). In this study, the serum total protein content in the group treated with 100 mg/kg EO and 1.5 mg/kg CA significantly decreased. This might be due to a decrease in the animal's protein synthesis capability or an increase in metabolism. Immunoglobulins (IgG and IgM) are important antibodies in animals that play a crucial role in the immune system. In this study, the IgG and IgM levels in Group D were significantly elevated, indicating the potential protective effects of the combination of plant essential oils and acidifiers on Xianjv chickens during their peak laying period. IL-2 is a pro-inflammatory cytokine that also plays a role in stimulating the animal's defense mechanisms. Its pleiotropic effects include the activation of B cells, T cells, and natural killer cells, as well as innate immune mechanisms associated with the function of phagocytes, such as macrophages and heterophils (Rochman, et al., 2009). IL-6 is produced by various cell types, including T cells, B cells, monocytes, macrophages, and fibroblasts. It plays a crucial role in both acute and chronic inflammatory responses. IL-6 can induce the production of acute phase proteins (such as C-reactive protein), promote the differentiation of B cells, and influence the activation and differentiation of T cells (Hirano, 2021). The results of this study show that the combination of 100 mg/kg EO and 1.5 mg/kg CA significantly reduces IL-2 and IL-6 levels in the serum of Xianjv chickens. This combination also helps alleviate the inflammatory response during the peak laying period of hens.

Oxidative stress refers to the imbalance between oxidation and antioxidant action in the body, leading to the production of various reactive oxygen species (ROS). This results in damage to proteins, nucleic acids, and lipids, causing tissue damage and disease progression. Antioxidant enzymes (SOD, CAT, GSH-Px, etc.) are the first line of defense against ROS, while MDA is the final product of lipid peroxidation and is commonly used as a biomarker to evaluate oxidative stress (Liu, et al., 2023; Zińczuk, et al., 2020). These are all important indicators reflecting the antioxidant status of animals. Xiao and colleagues found that essential oil supplements can significantly enhance the activity of superoxide dismutase and the overall antioxidant capacity in laying hens during the late laying period (Xiao, et al., 2022). The addition of mixed organic acids to the feed can enhance the antioxidant capacity of broilers (Ma, et al., 2021). In this study, supplementing with a low dose of compound acidifiers alone or in combination with plant essential oils significantly improved the antioxidant capacity of Xianjv chickens. However, supplementing the diet with a high dose of compound acidifiers further increased serum MDA levels. This may be due to the excessive supplementation of acidifiers disrupting the physiological functions of Xianjv chickens, thereby compromising their antioxidant status. There are reports in the literature that the use of organic acid mixtures alone can also lead to a decrease in the antioxidant capacity of ducks(Cao, et al., 2022). This is consistent with our findings. Therefore, we need to carefully evaluate the additive levels of organic acids in the feed and the ratio of their combination with plant essential oils to ensure that Xianjv chickens maintain optimal physiological conditions.

The growth, development, and reproduction of poultry depend on the secretion and metabolism of reproductive hormones, growth hormones, and other hormones. The normal function of the ovaries is crucial for maintaining the fertility and health of females. In theca cells of the ovary, LH (luteinizing hormone) stimulates the secretion of androgens, which are transferred to granulosa cells and converted into estradiol (E2) by aromatase. In granulosa cells, FSH (follicle-stimulating hormone) stimulates follicular development, while LH also plays a role in the development and maturation of the follicles (Bosch, et al., 2021). Growth hormone (GH) is associated with the onset of the laying cycle and the timing of ovulation in hens. In pre-hierarchical follicles, GH stimulates the theca layer to produce estradiol (E2), which further promotes the early stages of follicle recruitment and development (Hanlon, et al., 2020). Liu et al. found that the combined use of encapsulated *Bacillus subtilis* and essential oils can significantly increase the reproductive hormone levels in broiler breeders during the late laying period (Liu, et al., 2021). In this study, the group supplemented with 100 mg/kg EO and 1.5 g/kg CA significantly increased GH and E2 levels in Xianjv chickens, while FSH and LH levels significantly decreased. This indicates that the combined use of EO and CA helps Xianjv chickens enter the next egg-laying cycle earlier, which has the potential to improve their reproductive performance in the next cycle.

## Conclusions

In conclusion, the combined use of compound acidifiers (CA) and essential oils (EO) in the diet of Xianjv chickens can significantly improve egg quality. In addition, feeding a combination of 100 mg/kg EO and 1.5 g/kg CA significantly enhanced the intestinal digestive enzyme activity, antioxidant capacity, reduced inflammatory response, and improved immunity of Xianjv chickens. These findings support the potential of compound acidifiers and plant essential oils as effective alternatives to antibiotic growth promoters in poultry feeds, contributing to safer and more sustainable poultry production practices. However, the dosage and ratio of these additives need to be further optimized to ensure maximum benefit without compromising the physiological functions of the birds.

## Declaration of competing interest

We declare that we have no financial and personal relationships with other people or organizations that can inappropriately influence our work, there is no professional or other personal interest of any nature or kind in any product, service and/or company that could be construed as influencing the content of this paper.
